# Bivariate genome-wide association study identifies novel pleiotropic loci for lipids and inflammation

**DOI:** 10.1186/s12864-016-2712-4

**Published:** 2016-06-10

**Authors:** Symen Ligthart, Ahmad Vaez, Yi-Hsiang Hsu, Inflammation Working Group of the CHARGE Consortium, PMI-WG-XCP, LifeLines Cohort Study, Ronald Stolk, André G. Uitterlinden, Albert Hofman, Behrooz Z. Alizadeh, Oscar H. Franco, Abbas Dehghan

**Affiliations:** Department of Epidemiology, Erasmus University Medical Center, PO Box 2040, 3000CA Rotterdam, The Netherlands; Department of Epidemiology, University of Groningen, University Medical Center Groningen, Groningen, The Netherlands; Hebrew SeniorLife Institute for Aging Research and Harvard Medical School, Boston, MA USA; Molecular and Integrative Physiological Sciences Program, Harvard School of Public Health, Boston, MA USA; Framingham Heart Study, Framingham, MA USA; Department of Internal Medicine, Erasmus University Medical Center, Rotterdam, The Netherlands

**Keywords:** C-reactive protein, Inflammation, Lipids, Genome-wide association study, Genetic pleiotropy

## Abstract

**Background:**

Genome-wide association studies (GWAS) have identified multiple genetic loci for C-reactive protein (CRP) and lipids, of which some overlap. We aimed to identify genetic pleiotropy among CRP and lipids in order to better understand the shared biology of chronic inflammation and lipid metabolism.

**Results:**

In a bivariate GWAS, we combined summary statistics of published GWAS on CRP (*n* = 66,185) and lipids, including LDL-cholesterol, HDL-cholesterol, triglycerides, and total cholesterol (*n* = 100,184), using an empirical weighted linear-combined test statistic. We sought replication for novel CRP associations in an independent sample of 17,743 genotyped individuals, and performed *in silico* replication of novel lipid variants in 93,982 individuals. Fifty potentially pleiotropic SNPs were identified among CRP and lipids: 21 for LDL-cholesterol and CRP, 20 for HDL-cholesterol and CRP, 21 for triglycerides, and CRP and 20 for total cholesterol and CRP. We identified and significantly replicated three novel SNPs for CRP in or near *CTSB/FDFT1* (rs10435719, P_replication_: 2.6 × 10^−5^), *STAG1/PCCB* (rs7621025, P_replication_: 1.4 × 10^−3^) and *FTO* (rs1558902, P_replication_: 2.7 × 10^−5^). Seven pleiotropic lipid loci were replicated in the independent set of MetaboChip samples of the Global Lipids Genetics Consortium. Annotating the effect of replicated CRP SNPs to the expression of nearby genes, we observed an effect of rs10435719 on gene expression of *FDFT1*, and an effect of rs7621025 on *PCCB*.

**Conclusions:**

Our large scale combined GWAS analysis identified numerous pleiotropic loci for CRP and lipids providing further insight in the genetic interrelation between lipids and inflammation. In addition, we provide evidence for *FDFT1, PCCB* and *FTO* to be associated with CRP levels.

**Electronic supplementary material:**

The online version of this article (doi:10.1186/s12864-016-2712-4) contains supplementary material, which is available to authorized users.

## Background

Genome-wide association studies (GWAS) have identified hundreds of genetic loci for cardiovascular disease and it’s risk factors, including chronic inflammation and lipids [1–3]. Some of the identified genetic variants are associated with more than one phenotype, termed genetic pleiotropy [4]. Examples are *APOC1(rs4420638)* and *HNF1A (rs1183910),* which are associated both with lipids and C-reactive protein (CRP) [2, 3]. As randomized clinical trials have shown a coextending effect of statin treatment on the lowering of LDL-cholesterol and CRP, we do expect inflammation and lipids to share certain biological pathways [5, 6]. Moreover, there is accumulating evidence that the pleiotropic effects are partially independent, although the biological mechanisms are not fully understood [7]. The identification of further pleiotropic genes could provide insight into the biological mechanisms that link chronic inflammation to lipids.

Therefore, we aimed to identify further shared genes for lipids and CRP. In order to enhance the statistical power of genetic studies to find pleiotropic genes for the correlated phenotypes of interest, we applied a method that combines GWAS meta-analysis summary statistics allowing for mixed directions of effect, a common observed phenomenon in genetic pleitropy [8]. In a second step we sought to replicate novel associations with lipids and CRP in an independent sample of 93,982 genotyped individuals for lipids and 17,743 genotyped individuals for CRP. We identified multiple overlapping genetic variants between CRP and lipids and confirmed novel genes implicated in the biology of chronic inflammation.

## Results

### Bivariate genome-wide association analysis

We performed bivariate GWAS meta-analyses by combining summary statistics (Z test statistics) from the univariate GWAS of CRP pairing with the summary statistics of each GWAS of the lipid phenotypes, using an empirical-weighted linear-combined test statistics (eLC) [8]. This method allows mixed genetic effects in the univariate phenotype GWAS, a phenomenon commonly observed in genetic studies.

#### CRP and LDL-cholesterol

**Fig. 1:**
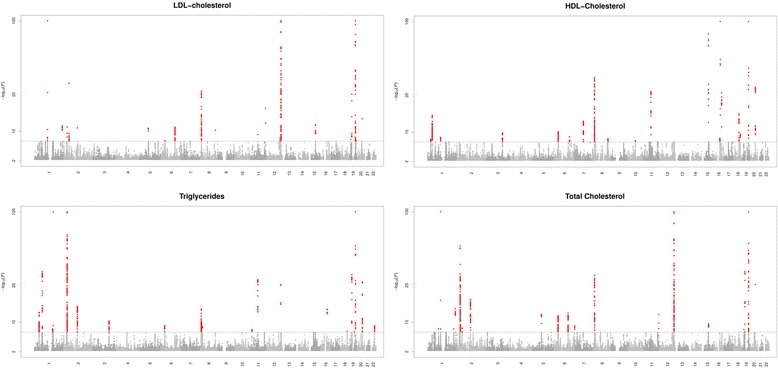
Manhattan Plots of the Bivariate Genome-Wide Association Studies Combining C-Reactive Protein with LDL-Cholesterol, HDL-Cholesterol, Triglycerides and Total Cholesterol

Manhattan plots for the bivariate GWAS are depicted in Fig. 1. Table 1 indicates the results from the bivariate analysis combining CRP and LDL-cholesterol genetic association data. The bivariate analysis resulted in 21 potentially pleiotropic loci. We identified fourteen loci associated with CRP levels which had no genome-wide significant SNP in the original GWAS of CRP. These potential novel associations were located in or near *CELSR2*, *IRF2BP2*, *ABCG8*, *GCNT4*, *HLA-DQB1*, *FRK*, *TRIB1*, *FADS2*, *ST3GAL4*, *BRAP*, *C12orf51*, *CARM1/LDLR*, *NCAN* and *RASIP1*. The potential novel associations for LDL-cholesterol were located in or near *GCKR*, *IL1F10*, *RORA*, *RASIP1* and in *HNF4A*. The SNPs identified in the bivariate GWAS near *HLA-DQB1*, *FRK*, *BRAP*, *c12orf51* and *CARM1/LDLR* were not genome-wide significant in the original univariate GWAS on LDL-cholesterol, however other SNPs in their vicinity were significant in the original GWAS on LDL-cholesterol and the loci have thus been reported previously. The variants in and near *PPP1R3B, HNF1A* and *APOC1* were already genome-wide significant in both GWAS of CRP and LDL-cholesterol.

**Table 1:** Results of Bivariate GWAS for C-Reactive Protein and LDL-Cholesterol Levels

SNP	Chromosome	Position	Effect Allele	C-reactive protein		LDL-cholesterol		Pleiotropy significance	Gene
				Beta	P-value	Beta	P-value		
rs646776	1	109620053	T	−0.018	0.02	0.171	4.5 × 10^−169^	4.3 × 10^−170^	*CELSR2*
rs661955	1	232909479	C	−0.021	1.7 × 10^−3^	0.034	1.2 × 10^−10^	3.2 × 10^−12^	*IRF2BP2*
rs3817588	2	27584716	T	0.053	1.8 × 10^−10^	0.024	4.2 × 10^−4^	6.4 × 10^−12^	*GCKR*
rs11887534	2	43919751	C	−0.049	2.5 × 10^−4^	−0.134	1.1 × 10^−31^	9.0 × 10^−33^	*ABCG8*
rs12711751	2	113554236	T	−0.044	1.6 × 10^−10^	0.014	4.8 × 10^−3^	1.2 × 10^−11^	*IL1F10*
rs4703642	5	74297918	A	0.018	3.0 × 10^−3^	−0.031	3.1 × 10^−10^	1.5 × 10^−11^	*GCNT4*
rs9275292	6	32771267	A	0.022	3.6 × 10^−4^	0.023	1.1 × 10^−5^	3.3 × 10^−8^	*HLA-DQB1*
rs3822857	6	116420624	C	−0.032	2.7 × 10^−6^	−0.030	2.3 × 10^−7^	7.6 × 10^−12^	*FRK*
rs9987289	8	9220768	A	−0.079	2.1 × 10^−12^	−0.071	2.0 × 10^−14^	2.3 × 10^−24^	*PPP1R3B*
rs8180991	8	126569532	C	−0.026	9.0 × 10^−4^	−0.041	8.0 × 10^−10^	5.1 × 10^−11^	*TRIB1*
rs174574	11	61356918	A	−0.027	1.7 × 10^−3^	−0.050	1.1 × 10^−8^	7.8 × 10^−10^	*FADS2*
rs11220463	11	125753421	A	0.032	2.8 × 10^−3^	−0.070	1.3 × 10^−15^	5.8 × 10^−17^	*ST3GAL4*
rs10744775	12	110580598	T	0.021	4.0 × 10^−3^	−0.030	5.3 × 10^−7^	3.1 × 10^−8^	*BRAP*
rs2285810	12	111183923	T	0.019	6.8 × 10^−3^	−0.030	8.3 × 10^−8^	8.3 × 10^−9^	*C12orf51*
rs1183910	12	119905190	A	−0.151	4.6 × 10^−113^	0.042	5.8 × 10^−15^	5.6 × 10^−128^	*HNF1A*
rs340005	15	58665322	A	0.044	3.2 × 10^−11^	−0.015	3.4 × 10^−3^	1.7 × 10^−12^	*RORA*
rs1529711	19	10884434	T	0.030	8.4 × 10^−4^	0.037	1.5 × 10^−6^	1.5 × 10^−8^	*CARM1/LDLR*
rs2228603	19	19190924	T	0.036	2.9 × 10^−3^	−0.089	1.4 × 10^−19^	6.5 × 10^−21^	*NCAN*
rs4420638	19	50114786	A	0.240	1.0 × 10^−129^	−0.215	8.7 × 10^−147^	1.2 × 10^−283^	*APOC1*
rs2287921	19	53920084	T	−0.019	3.6 × 10^−3^	−0.026	3.4 × 10^−7^	2.8 × 10^−8^	*RASIP1*
rs1800961	20	42475778	T	−0.120	2.4 × 10^−11^	−0.070	2.4 × 10^−5^	3.8 × 10^−14^	*HNF4A*

*Abbreviations*: *SNP* single nucleotide polymorphism

For both CRP and the lipid phenotype, the effect estimates are according to the original GWAS

Chromosome and position are in NCBI genome build 36

Beta coefficient for CRP represents 1-unit change in the natural log–transformed CRP (mg/L) per copy increment in the coded allele

Beta coefficient for LDL-cholesterol represents 1-unit change in the standardized LDL-cholesterol levels per copy increment in the coded allele

#### CRP and HDL-cholesterol

We identified 20 potential pleiotropic SNPs (Table 2)*.* The variants near *CELSR2, STAG1, HLA-DRA, JMJD1C, FADS1, LIPC, CETP, LYPLA3, LIPG* and *MC4R* were not genome-wide significant in the original CRP meta-GWAS analysis. Seven SNPs were potentially novel for both CRP and HDL-cholesterol: the SNP rs12742376 located in *C1orf172* on chromosome 1 (*P*_bivariate_ = 1.4 × 10^−8^), rs7621025 in *STAG1* on chromosome 3 (*P*_*bivariate*_ = 1.2 × 10^−9^), rs9378212 near *HLA-DRA* (*P*_*bivariate*_ = 6.7 × 10^−10^), rs10761731 in *JMJD1C* (*P*_*bivariate*_ = 2.2 × 10^−8^), rs1936797 in *RSPO3* on chromosome 6 (*P*_bivariate_ = 6.7 × 10^−9^), rs4871137 near *SNTB1* (*P*_bivariate_ = 3.3 × 10^−8^) on chromosome 8 and the *FTO* SNP rs1558902 (*P*_bivariate_ = 5.0 × 10^−9^) on chromosome 16. The variants near *CELSR2* and *PLTP* were not significant in the original GWAS on HDL-cholesterol, but these loci were identified in the original GWAS. The variants in or near *PABPC4, BAZ1B, PPP1R3B, APOC1* and *HNF4A* were already genome-wide significant in both the CRP and HDL-cholesterol univariate GWAS.

**Table 2:** Results of Bivariate GWAS Analyses for C-Reactive Protein and HDL-Cholesterol Levels

SNP	Chromosome	Position	Effect Allele	C-reactive protein		HDL-cholesterol		Pleiotropy significance	Gene
				Beta	P-value	Beta	P-value		
rs12742376	1	27157782	T	−0.027	1.7 × 10^−2^	−0.046	2.8 × 10^−7^	1.4 × 10^−8^	*C1orf172*
rs4660293	1	39800767	A	−0.044	1.2 × 10^−9^	0.034	4.0 × 10^−10^	3.1 × 10^−15^	*PABPC4*
rs646776	1	109620053	T	−0.018	1.8 × 10^−2^	−0.033	6.4 × 10^−8^	3.2 × 10^−9^	*CELSR2*
rs7621025	3	137754936	T	0.028	1.7 × 10^−4^	0.026	4.1 × 10^−6^	1.2 × 10^−9^	*STAG1*
rs9378212	6	32553669	T	0.027	4.9 × 10^−5^	0.021	8.1 × 10^−6^	6.7 × 10^−10^	*HLA-DRA*
rs1936797	6	127474350	A	0.022	2.8 × 10^−3^	0.022	9.9 × 10^−7^	6.7 × 10^−9^	*RSPO3*
rs13244268	7	72549779	T	0.054	2.6 × 10^−8^	−0.045	1.3 × 10^−9^	1.2 × 10^−13^	*BAZ1B*
rs9987289	8	9220768	A	−0.079	2.1 × 10^−12^	−0.083	6.4 × 10^−25^	1.2 × 10^−39^	*PPP1R3B*
rs4871137	8	121937732	T	−0.021	2.2 × 10^−3^	−0.026	5.6 × 10^−6^	3.3 × 10^−8^	*SNTB1*
rs10761731	10	64697616	A	0.023	2.7 × 10^−4^	−0.025	2.5 × 10^−7^	2.2 × 10^−8^	*JMJD1C*
rs174546	11	61326406	T	−0.017	1.2 × 10^−2^	−0.048	2.6 × 10^−22^	1.6 × 10^−24^	*FADS1*
rs1077834	15	56510771	T	−0.016	4.0 × 10^−2^	−0.114	9.6 × 10^−84^	2.5 × 10^−87^	*LIPC*
rs1558902	16	52361075	A	0.032	2.0 × 10^−6^	−0.021	4.6 × 10^−6^	5.0 × 10^−9^	*FTO*
rs711752	16	55553712	A	0.016	1.8 × 10^−2^	0.192	2.1 × 10^−297^	4.3 × 10^−308^	*CETP*
rs17688076	16	66843928	A	0.019	4.9 × 10^−2^	0.070	3.9 × 10^−22^	1.8 × 10^−23^	*LYPLA3*
rs11874381	18	45457406	A	0.013	4.9 × 10^−2^	0.038	1.2 × 10^−14^	1.0 × 10^−15^	*LIPG*
rs12967135	18	56000003	A	0.029	1.2 × 10^−4^	−0.036	6.6 × 10^−9^	4.3 × 10^−10^	*MC4R*
rs4420638	19	50114786	A	0.240	1.0 × 10^−129^	0.071	4.4 × 10^−21^	2 × 10^−164^	*APOC1*
rs1800961	20	42475778	T	−0.120	2.4 × 10^−11^	−0.129	1.1 × 10^−15^	3.9 × 10^−28^	*HNF4A*
rs6065906	20	43987422	T	0.036	5.9 × 10^−6^	0.058	1.9 × 10^−22^	5.1 × 10^−29^	*PLTP*

*Abbreviations*: SNP single nucleotide polymorphism

For both CRP and the lipid phenotype, the effect estimates are according to the original GWAS

Chromosome and position are in NCBI genome build 36

β coefficient for CRP represents 1-unit change in the natural log–transformed CRP (mg/L) per copy increment in the coded allele

Beta coefficient for HDL-cholesterol represents 1-unit change in the standardized HDL-cholesterol levels per copy increment in the coded allele

#### CRP and Triglycerides

Table 3 lists the 21 potentially pleiotropic SNPs that were identified combining the GWAS results of triglycerides and CRP. For triglycerides, we identified eleven potential novel associations compared to the original GWAS located in or near *PABPC4, LEPR, ADAR, CRP, IL1F10, PPP1R3B, CTSB/FDFT1, ARNTL, CABP1, MC4R* and *HPN*. The variant near *PLA2G6* was not genome-wide significant in the original GWAS, but this locus was identified in the original GWAS. The variants in and near *ADAR, MSL2L1, HLA-C, CTSB/FDFT1, LPL, ARNTL, FADS1, CETP, MC4R, SF4, HPN, ZNF335/PLTP* and *PLA2G6* were potential novel associations with CRP level. Five loci were not genome-wide significant in either the original GWAS on CRP or triglycerides: the SNP rs1127311 within *ADAR* on chromosome 1 (*P*_*bivariate*_ *=* 6.4 × 10^−9^), rs10435719 located 77Kb upstream of *CTSB* on chromosome 8 (*P*_*bivariate*_ *=* 2.0 × 10^−10^), rs10832027 located in the second intron of *ARNTL* on chromosome 11 (*P*_*bivariate*_ *=* 9.4 × 10^−9^), rs571312 on chromosome 18 near *MC4R* (*P*_*bivariate*_ *=* 2.8 × 10^−8^)*,* and the chromosome 19 rs1688043 in the fifth intron of *HPN* (*P*_*bivariate*_ *=* 4.1 × 10^−8^). In both the original GWAS of CRP and triglycerides, *GCKR* and *APOC1* were already genome-wide significant.

**Table 3:** Results of Bivariate GWAS Analyses for C-Reactive Protein and Triglycerides Levels

SNP	Chromosome	Position	Effect Allele	C-reactive protein		Triglycerides		Pleiotropy significance	Gene
				Beta	P-value	Beta	P-value		
rs4660808	1	39791096	T	0.046	8.6 × 10^−10^	0.028	3.1 × 10^−7^	2.2 × 10^−13^	*PABPC4*
rs11208722	1	65943589	A	−0.083	1.2 × 10^−32^	0.012	0.02	8.1 × 10^−36^	*LEPR*
rs1127311	1	152823287	A	−0.031	9.3 × 10^−7^	0.012	5.5 × 10^−3^	6.4 × 10^−9^	*ADAR*
rs12755606	1	157936960	C	−0.153	3.0 × 10^−112^	0.012	0.01	4.0 × 10^−120^	*CRP*
rs1260326	2	27584444	T	0.089	1.7 × 10^−42^	0.116	5.7 × 10^−133^	4.4 × 10^−151^	*GCKR*
rs13409360	2	113554573	A	0.048	1.3 × 10^−12^	−0.013	8.6 × 10^−3^	5.3 × 10^−15^	*IL1F10*
rs645040	3	137409312	T	−0.023	2.5 × 10^−3^	0.030	2.5 × 10^−8^	4.6 × 10^−11^	*MSL2L1*
rs2524163	6	31367558	T	0.025	1.5 × 10^−4^	0.027	1.7 × 10^−8^	7.9 × 10^−10^	*HLA-C*
rs9987289	8	9220768	A	−0.079	2.1 × 10^−12^	0.020	0.02	2.9 × 10^−14^	*PPP1R3B*
rs10435719	8	11814313	T	0.026	7.6 × 10^−5^	−0.022	4.1 × 10^−6^	2.0 × 10^−10^	*CTSB*
rs1441759	8	19909843	C	0.11	3.3 × 10^−4^	0.125	2.1 × 10^−8^	2.0 × 10^−9^	*LPL*
rs10832027	11	13313759	A	0.032	8.5 × 10^−7^	0.020	1.1 × 10^−4^	9.4 × 10^−9^	*ARNTL*
rs174546	11	61326406	T	−0.017	0.01	0.048	5.4 × 10^−24^	5.2 × 10^−27^	*FADS1*
rs2686555	12	119579555	A	−0.059	1.7 × 10^−19^	0.010	0.03	1.6 × 10^−21^	*CABP1*
rs11508026	16	55556829	T	0.014	0.03	−0.038	1.3 × 10^−12^	3.1 × 10^−14^	*CETP*
rs571312	18	55990749	A	0.033	3.5 × 10^−5^	0.026	1.2 × 10^−5^	2.8 × 10^−8^	*MC4R*
rs10401969	19	19268718	T	−0.031	0.02	0.112	1.6 × 10^−29^	1.6 × 10^−32^	*SF4*
rs1688043	19	40245181	T	−0.038	2.4 × 10^−3^	0.037	1.2 × 10^−5^	4.1 × 10^−8^	*HPN*
rs4420638	19	50114786	A	0.24	1.0 × 10^−129^	−0.068	5.4 × 10^−22^	1.7 × 10^−171^	*APOC1*
rs4465830	20	44018827	A	0.036	7.0 × 10^−6^	−0.050	2.0 × 10^−17^	2.0 × 10^−24^	*ZNF335/PLTP*
rs2277844	22	36907461	A	−0.018	5.7 × 10^−3^	0.025	1.5 × 10^−7^	9.2 × 10^−10^	*PLA2G6*

*Abbreviations*: *SNP* single nucleotide polymorphism

For both CRP and the lipid phenotype, the effect estimates are according to the original GWAS

Chromosome and position are in NCBI genome build 36

β coefficient for CRP represents 1-unit change in the natural log–transformed CRP (mg/L) per copy increment in the coded allele

Beta coefficient for triglycerides represents 1-unit change in the standardized triglyceride levels per copy increment in the coded allele

#### CRP and total cholesterol

Twenty potentially pleiotropic SNPs were identified combining CRP and total cholesterol (Table 4). The SNPs in or near *ZNF644*, *SLC44A4*, *C7orf50* and *RORA* were potentially novel for total cholesterol. The variants near *HLX, ABCG5, IL1F10, C7orf60* and *CARM1* were not genome-wide significant in the GWAS on total cholesterol, but the loci were identified in this original GWAS. For CRP, *ZNF664*, *CELSR2*, *HLX*, *IRF2BP2*, *ABCG5*, *GCNT4*, *SLC44A4*, *HLA-DQB1*, *FRK*, *ST3GAL4*, *CARM1* and *NCAN* were potentially novel compared to the univariate GWAS. The SNPs near *ZNF644* and *C7orf50* were novel pleiotropic loci for both CRP and total cholesterol.

**Table 4:** Results of Bivariate GWAS Analyses for C-Reactive Protein and Total Cholesterol Levels

SNP	Chromosome	Position	Effect Allele	C-reactive protein		Total cholesterol		Pleiotropy significance	Gene
				Beta	P-value	Beta	P-value		
rs469772	1	91302893	T	−0.042	1.6 × 10^−7^	−0.020	1.5 × 10^−3^	1.5 × 10^−8^	*ZNF644*
rs629301	1	109619829	T	−0.017	2.8 × 10^−2^	0.149	5.8 × 10^−131^	5.7 × 10^−132^	*CELSR2*
rs17597773	1	219121384	C	0.020	7.5 × 10^−3^	−0.031	7.1 × 10^−8^	6.6 × 10^−9^	*HLX*
rs661955	1	232909479	C	−0.021	1.7 × 10^−3^	0.036	1.0 × 10^−12^	2.2 × 10^−14^	*IRF2BP2*
rs1260326	2	27584444	T	0.089	1.7 × 10^−42^	0.055	7.3 × 10^−27^	2.6 × 10^−63^	*GCKR*
rs4148191	2	43896408	A	−0.050	2.5 × 10^−4^	−0.054	1.1 × 10^−6^	3.7 × 10^−09^	*ABCG5*
rs6734238	2	113557501	A	−0.047	4.8 × 10^−13^	0.023	1.2 × 10^−5^	5.8 × 10^−17^	*IL1F10*
rs4703642	5	74297918	A	0.018	3.0 × 10^−3^	−0.033	2.0 × 10^−11^	7.3 × 10^−13^	*GCNT4*
rs577272	6	31945942	A	0.020	1.1 × 10^−3^	0.026	2.3 × 10^−7^	1.6 × 10^−8^	*SLC44A4*
rs2858310	6	32776301	A	0.026	8.7 × 10^−5^	0.033	3.3 × 10^−10^	3.8 × 10^−12^	*HLA-DQB1*
rs3822857	6	116420624	C	−0.032	2.7 × 10^−6^	−0.033	4.7 × 10^−9^	2.1 × 10^−12^	*FRK*
rs6951245	7	1024719	A	0.03	5.5 × 10^−4^	0.037	6.1 × 10^−8^	2.6 × 10^−9^	*C7orf50*
rs2126259	8	9222556	T	−0.072	5.7 × 10^−12^	−0.085	9.0 × 10^−24^	1.4 × 10^−31^	*PPP1R3B*
rs11220463	11	125753421	A	0.032	2.8 × 10^−3^	−0.057	2.1 × 10^−11^	7.3 × 10^−13^	*ST3GAL4*
rs1183910	12	119905190	A	−0.151	4.6 × 10^−113^	0.040	5.2 × 10^−14^	8.2 × 10^−128^	*HNF1A*
rs340025	15	58695599	T	−0.036	8.3 × 10^−9^	0.015	2.4 × 10^−3^	2.5 × 10^−10^	*RORA*
rs1529711	19	10884434	T	0.030	8.4 × 10^−4^	0.038	6.3 × 10^−7^	3.4 × 10^−8^	*CARM1*
rs2228603	19	19190924	T	0.036	2.9 × 10^−3^	−0.118	4.3 × 10^−34^	1.1 × 10^−35^	*NCAN*
rs4420638	19	50114786	A	0.240	1.0 × 10^−129^	−0.184	5.2 × 10^−111^	3.8 × 10^−249^	*APOC1*
rs1800961	20	42475778	T	−0.120	2.4 × 10^−11^	−0.118	5.7 × 10^−13^	1.0 × 10^−20^	*HNF4A*

*Abbreviations*: *SNP* single nucleotide polymorphism

For both CRP and the lipid phenotype, the effect estimates are according to the original GWAS

Chromosome and position are in NCBI genome build 36

Beta coefficient for total cholesterol represents 1-unit change in the standardized total cholesterol levels per copy increment in the coded allele

β coefficient for CRP represents 1-unit change in the natural log–transformed CRP (mg/L) per copy increment in the coded allele

### Replication of the novel pleiotropic loci

In total, we sought replication for 36 potential novel SNPs for CRP in 17,743 genotyped individuals from three independent cohort studies. Using a Bonferroni corrected threshold for multiple testing (0.05/36 = 1.4 × 10^−3^), three SNPs remained significantly associated with CRP levels when we performed replication analysis (Additional file 1: Table S1). These variants included the SNPs rs10435719 in *CTSB/FDFT1* (*P*_*replication*_ *=* 2.6 × 10^−5^)*,* rs1558902 near FTO (*P*_*replication*_ *=* 2.7 × 10^−5^) and rs7621025 near *STAG1* (*P*_*replication*_ *=* 1.4 × 10^−3^).

We aimed replication for 23 potential novel SNPs for lipids (4 for LDL-cholesterol, 7 for HDL-cholesterol, 9 for triglycerides and 3 for total cholesterol) in an *in silico* analysis including 93,982 individuals. We could significantly replicate 2 variants for LDL-cholesterol (*HNF4A* and *RASIP1*), three for HDL-cholesterol (*C1orf172, RSPO3* and *STAG1),* one for triglycerides (*CTSB*) and one for total cholesterol (*C7orf50*) (Additional file 1: Table S2).

### Expression Quantitative Trait Loci (eQTL)

To annotate the effect of the replicated pleiotropic variants to the expression level of nearby genes, we investigated the association between the pleiotropic variants and gene expression levels in three different tissues relevant to CRP and lipids by use of large publicly available datasets: whole blood (*N* = 5311) [9], liver (*N* = 427 [10] and 266 [11]) and adipose tissue [12] (*N* = 111). For the replicated pleiotropic variant rs10435719 near *CTSB* and *FDFT1*, we observed significant associations in whole blood with expression levels of two genes: *CTSB* itself (*P* = 1.67 × 10^−6^), and *FDFT1* (*P* = 1.10 × 10^−96^). In addition, the SNP rs7621025 near *STAG1* and *PCCB* was strongly associated with expression of the gene *PCCB* in whole blood (*P* = 1.1 × 10^−40^). No eQTL effect was observed in the liver and adipose tissue.

## Discussion

We identified fifty potential pleiotropic SNPs which affect both CRP and lipid levels, of which we replicated three novel CRP variants: rs10435719 (*CTSB/FDFT1*), rs7621025 (*STAG1/PCCB*) and rs1558902 (*FTO*). In silico expression analyses suggested a role for rs10435719 in the gene expression of both *CTSB* and *FDFT1* and rs7621025 appeared to have an effect on the gene expression of *PCCB*.

The locus harboring rs10435719 near *CTSB* and *FDFT1* that was identified for CRP in our study has previously been identified for triglycerides in the joint analysis of the Global Lipids Genetics Consortium combining GWAS data with Metabochip association results [13]*.* We observed a significant effect of rs10435719 on the expression of both *CTSB* and *FDFT1*. The effect of the CRP increasing allele (T) was weakly associated with a decrease in the expression of *CTSB,* whilst we observed a strong association of the T-allele with an increase of *FDFT1* gene expression*. FDFT1* encodes the enzyme squalene synthase which is involved in the cholesterol biosynthesis [14]. Apart from lipids, *FDFT1* has been identified in a GWAS on fatty liver disease [15]. Squalene Synthase Inhibitors (SSI) have been developed and are successful in the reduction of cholesterol levels as well as CRP levels [16]. This pleiotropic effect of cholesterol synthesis blockers on both lipid levels and inflammation is thought to be the consequence of altered isoprenoids levels that may activate pro-inflammatory pathways [17]. The observation that the CRP increasing allele is associated with an increase in *FDFT1* gene expression suggests an effect of rs10435719 on serum CRP through *FDFT1.* However, we searched in large databases to identify robust eQTL effects of the novel variants. Therefore, we were unable to test the association between the expression and CRP and we cannot draw a firm conclusion on the causal effect of the gene expression in the association between the genetic variant and CRP.

We identified the SNP rs7621025 (*STAG1/PCCB*) as a pleiotropic variant for HDL-cholesterol and CRP. We confirmed the effect of rs7621025 on serum CRP in an independent set of individuals and this genomic region has been identified in a GWAS of lipids [13]. The SNP rs7621025 is located within *STAG1*, but has a strong effect on the expression of *PCCB*, located ±300 kb downstream of rs7621025 on chromosome 3. *PCCB* has been identified in a GWAS of the protein fibrinogen, an acute phase response protein sharing many genes with CRP [18]. Our results provide further evidence that the *PCCB* gene is involved in inflammation.

We identified the *FTO* gene as a pleiotropic locus for CRP and HDL-cholesterol. The A allele of rs1558902 was associated with an increase of CRP and a decrease in HDL cholesterol. In several GWAS on BMI, the A allele of rs1558902 was also associated with an increase in BMI [19, 20]. Previous studies have highlighted the causal effect of obesity on inflammation [21], and the effect directions are consistent with mediation of both the association with CRP and HDL-cholesterol by BMI. We have previously shown that the effect of FTO on CRP is indeed mediated through BMI [22]. Further research is needed to demonstrate whether this is also true for HDL-cholesterol. Our results provide further evidence for the role of obesity in inflammation and highlight the pleiotropic effects of the *FTO* locus on both chronic inflammation and lipid metabolism.

Genetic pleiotropy can be divided in biological and mediated pleiotropy [4]. In biological pleiotropy, the effect of the pleiotropic variant on two or more phenotypes is independent. In mediated pleiotropy, one phenotype mediates the association between the genetic variant and the second phenotype. Both biological and mediated pleiotropic effects may occur for CRP and lipids [23]. In the current study, we did not disentangle the different subtypes of pleiotropy. Moreover, we observed pleiotropic variants with an opposite direction of effect than expected based on the phenotypical correlation in observational epidemiological studies. In biological pleiotropy, opposite directions of effect may occur. As an example, although CRP and LDL-cholesterol are positively associated in observational epidemiological studies, the A-allele of the SNP rs1183910 (*HNF1A*) is associated with lower CRP levels but higher LDL-cholesterol. Opposite direction of effects are often seen in genetic studies and highlight the complex interplay between correlated phenotypes, in our study CRP and lipids [20]. We did not disentangle the different subtypes of pleiotropy, which is a limitation of the current study.

Our study has certain strengths. We add to previous studies showing that the multivariate method we applied can be effectively utilized to identify potential novel and pleiotropic loci. This method only requires GWAS summary data instead of individual level data from all participating cohorts. Thanks to close collaboration between studies across the world, researchers have performed large GWAS meta-analyses for a vast amount of phenotypes and this data is available for further research. Second, we used the largest GWAS meta-analyses that have so far been done on CRP and lipid levels to identify pleiotropic genetic loci. By doing so, we enhanced the statistical power to detect these loci considerably. Third, we provided robust evidence for three novel CRP loci by replication in an independent sample of genotyped individuals. A limitation of the bivariate meta-analysis is that very strong signals in one of the individual traits may overshadow the weak association with the other phenotype. We set a criterion for the univariate p-values <0.05 to minimize the chance of false positive findings. In many instances the effect of the pleiotropic loci on CRP or lipids is very small. We did not replicate all our pleiotropic loci. This could be due to lack of power in the replication. In concordance, we replicated a larger proportion of the lipid variants in the larger lipid replication sample compared to CRP. Also, variants closer to significance did replicate in the replication study of both CRP and lipids. Also, several variants had substantial heterogeneity I^2^ in the replication which lowers the power for replication. Furthermore, the replication sample size was for some variants smaller than 17,743 due to absence of the variants in one or more of the replication studies. However, we cannot rule out the possibility that bivariate p-values are driven by strong associations with one of the phenotypes and produce false positive results. In addition, for the replication of the lipid variants, we used the Metabochip results from the GLGC. Several variants selected for replication were not present on the Metabochip. Although we selected the best available proxy SNP for replication, variants in moderate LD may have limited power for replication. The method used in the current manuscript to prioritize variants with pleiotropic effects among inflammation and cholesterol are hypothesis generating and further functional work regarding the role of the identified variants in cholesterol metabolism and inflammation is necessary.

## Conclusions

Our results provide evidence for substantial overlap in genetic susceptibility for chronic inflammation and lipid metabolism. In addition, through bivariate genome-wide association studies and replication in an independent sample of individuals we could identify novel genes for CRP.

## Methods

The present study includes three stages. First, we performed a bivariate GWAS combining published GWAS data on CRP and lipids to identify pleiotropic variants for CRP and lipids. In a second step, we sought replication of novel associations in independent samples of genotyped individuals. Finally, we carried out functional analyses in a third step to point out potential underlying transcriptional mechanisms.

We used the data from the largest published GWAS on CRP as well as the publically available GWAS on lipids from GLGC to explore the genetic pleiotropy between inflammation and lipids [2, 3]. We combined summary association test statistics from the CRP GWAS separately with the GWAS on HDL-cholesterol, LDL-cholesterol, triglycerides and total cholesterol. The CRP GWAS meta-analysis included 65,000 individuals from 15 different studies in the discovery panel and after replication, 18 loci were genome-wide significantly associated with serum CRP level [3]. The lipids GWAS comprised 100,184 individuals for total cholesterol, 95,454 for LDL-cholesterol, 99,900 for HDL-cholesterol and 96,598 for triglycerides across 46 studies. The lipid GWAS identified a total of 95 lipid loci (52 for total cholesterol, 37 for LDL-cholesterol, 47 for HDL-cholesterol and 32 for triglycerides) [2]. The CRP and lipids GWAS used HapMap imputed data (build 36). All studies that contributed genotype data to the CRP GWAS also contributed data to the lipids GWAS. We ensured that effect alleles were harmonized across the two GWAS before applying the bivariate GWAS method. Overall, 2,501,549 common Single Nucleotide Polymorphisms (SNPs) were tested for their association with CRP and total cholesterol, 2,501,711 with CRP and triglycerides, 2,501,543 with CRP and HDL-cholesterol and 2,501,749 with CRP and LDL-cholesterol. An aggregated p-value was calculated using the method described below.

### Bivariate genome-wide association study

To better understand the shared biology of CRP and lipids by further identifying shared genes between CRP and lipids, we aimed to increase power by combining the summary statistics from the CRP and lipid GWAS. We chose to use a recently introduced method that performs bivariate GWAS allowing for mixed directions of effect. The method combines summary statistics (Z test statistics) from univariate GWAS of CRP pairing with the summary statistics of each univariate GWAS meta-analysis of lipid phenotypes, using an empirical-weighted linear-combined test statistics (eLC), implemented in a C++ eLX package. We have recently used this method in the identification of pleiotropic genes for menopause and menarche and the details of the method are presented elsewhere. [8, 24]. eLC allows having opposite direction of effect on the combined phenotypes, which is common between CRP and cholesterol phenotypes [2, 3]. Briefly, eLC directly combines correlated Z test statistics (calculated as *β*/*SE* derived from the original GWAS) obtained from univariate GWAS meta-analyses with a weighted sum of univariate test statistics to empirically maximize the overall association signals and also to account for the phenotypical correlations among CRP and lipids. Our eLC approach is expressed as

$$ {S}_{eLC}={\displaystyle \sum_1^k\left[ \max {\left(\left|{T}_k\right|,c\right)}^{*}\left|{T}_k\right|\right]} $$

where T_k_ is a matrix of K statistics for K phenotypes (for bivariate, K is equal to 2) and c is a given non-negative constant. The optimal weighting is estimated empirically using the Monte Carlo Simulation [25] and the bona-fide p-values for eLC test statistics are calculated through permutation. The sample covariance matrix of the test statistics of all SNPs from the univariate GWAS analyses is used as an approximation of the variance-covariance matrix **Σ** of univariate test statistics. **Σ**:

$$ \left[\begin{array}{cc}\hfill \mathrm{V}\mathrm{a}\mathrm{r}\left({\mathrm{Z}}_1\right)\hfill & \hfill \mathrm{C}\mathrm{o}\mathrm{v}\left({\mathrm{Z}}_1,{\mathrm{Z}}_2\right)\hfill \\ {}\hfill \mathrm{C}\mathrm{o}\mathrm{v}\left({\mathrm{Z}}_1,{\mathrm{Z}}_2\right)\hfill & \hfill \mathrm{V}\mathrm{a}\mathrm{r}\left({\mathrm{Z}}_2\right)\hfill \end{array}\right] $$

where *Z*_*1*_ and *Z*_*2*_ consist of unbiased univariate test statistics of all the SNPs for the two traits on genome-wide scale for the first (Z_1_) and second (Z_2_) trait*.* The null hypothesis in the bivariate analysis is β_1 = 0 AND β_2 = 0; the H1 is β_1 not equal to 0 or β_2 not equal to 0. The results were considered genome-wide significant when (1) the bivariate p-values were < 5 × 10^−8^ and (2) the bivariate p-value was at least one order of magnitude lower than both individual trait p-values and (3) when the individual trait p-values were at least nominally significant (p-value < 0.05). When multiple SNPs were significant in a locus, the SNP with the lowest p-value was chosen for replication. The eLC method is implemented in eLX package using C++ (see Weblinks).

### Replication study

The bivariate GWAS resulted in three possible scenarios. First, the pleiotropic variant or the locus harboring the pleiotropic variant (defined as ±500 MB of the pleiotropic SNP) was genome-wide significant in both the primary univariate GWAS of CRP and the lipid trait. Second, the pleiotropic signal was significant in either the CRP or the lipid univariate GWAS. Third, the pleiotropic signal was neither genome-wide significant in the CRP nor in the lipid GWAS. Per definition, a variant is considered pleiotropic when there is robust evidence for an association with two or more phenotypes. Therefore, we only selected the variants that were not genome-wide significant in the primary univariate GWAS for replication in an independent sample of genotyped samples. We intended to replicate the novel associations with CRP levels in three cohort studies that did not contribute to the original CRP GWAS. The independent cohorts were the second (*n* = 1943) and third (*n* = 2962) cohort of the Rotterdam Study and the LifeLines cohort study (*n* = 12,838; Additional file 1) [6, 7, 26, 27]. The total sample size for the replication of potentially novel CRP variants comprised 17,743 individuals. In an attempt to replicate the potential novel lipid variants, we performed an in silico replication in the publicly available association results from the participants of the GLGC that did not contribute to the original lipids GWAS we used for the pleiotropy analysis. This replication set comprises 93,982 individuals genotyped using the Metabochip array [13, 28]. For the SNPs that were not available on the Metabochip, we selected the best available proxy SNP on the Metabochip for replication (*r*^*2*^ > 0.5). We used a Bonferroni corrected p-value of 0.05 divided by the number of SNPs tested for replication as a threshold of significance in the replication study.

### Ethics, consent and permissions

All participants of the Rotterdam and Lifelines study provided written informed consent.

### Expression Quantitative Trait Loci (eQTL)

In an attempt to annotate the pleiotropic variants to a pleiotropic gene, we searched in tissues related to lipids and inflammation for eQTL effects of the pleiotropic variants or reasonable proxy variants (*r*^*2*^ > 0.80).

The eQTL analyses in whole blood comprised 5311 individuals from seven studies in the discovery setting with both genetic and gene expression data available [9]. The discovery meta-analysis including the seven studies (EGCUT, InCHIANTI, Rotterdam Study, Fehrmann, HVH, SHIP-TREND and DILGOM). Results are publicly available (access URL: http://genenetwork.nl/bloodeqtlbrowser/). eQTLs were deemed cis when the distance between the SNP and the midpoint of the RNA probe was <250 kb. We only considered a significant eQTL effect of the pleiotropic SNP when the p-value exceeded the FDR corrected threshold for multiple testing.

We searched for liver eQTL effects by use of the eQTL browser provided by the university of Chicago (access URL: http://eqtl.uchicago.edu/cgi-bin/gbrowse/eqtl/). The liver tissue dataset by Schadt et al. comprised 427 individuals from European ancestry with liver specific gene expression and genotyping data available [10]. An eQTL was deemed cis when the SNP was within 1 Mb of the annotated start or stop site of the corresponding structural gene. The authors used an FDR correction of 10 % for a significant association. The dataset by Innocenti et al. comprised 266 individuals from 2 different studies. Cis eQTL was defined as <250 kb from the gene transcription start site and the FDR for significant association was set to 5 % [11].

We used the GTEx adipose tissue dataset (access URL: http://www.gtexportal.org/home/eqtls/tissue?tissueName=Adipose_Subcutaneous) to search for potential eQTLs in adipose tissue. The dataset consisted of 111 individuals with both gene expression and genotype data available [12] Cis radius was defined as +/- 1mb from transcription start site. A eQTL was deemed significant when the FDR q-value < =5 %.

## Additional file

Additional file 1: Study-specific Methods Section for the Replication Section. **Table S1.** Replication Results for C-Reactive Protein. **Table S2.** Replication Results for lipids. **Table S3.** Proxy variants for the Single Nucleotide Polymophisms not Available on the Metabochip Array. (DOCX 30 kb)

## Abbreviations

CRP: C-reactive protein
GWAS: genome-wide association study
