# Supplementary material for: Bivariate genome-wide association study identifies novel pleiotropic loci for lipids and inflammation
Source: BMC Genomics. 2016 Jun 10;17:443. doi: 10.1186/s12864-016-2712-4 (PMC4901478; doi:10.1186/s12864-016-2712-4)
Supplement: Additional file 1: — Study-specific Methods Section for the Replication Section. Table S1. Replication Results for C-Reactive Protein. Table S2. Replication Results for lipids. Table S3. Proxy variants for the Single Nucleotide Polymophisms not Available on the Metabochip Array. (DOCX 30 kb) [file 12864_2016_2712_MOESM1_ESM.docx]

**Supplemental Material**

Supplement to: Bivariate Genome-Wide Association Study Identifies Novel Pleiotropic Loci for Lipids and Inflammation. Symen Ligthart, Ahmad Vaez, Yi-Hsiang Hsu et al.

**Bivariate Genome-Wide Association Study Identifies Novel Pleiotropic Loci for Lipids and Inflammation.**

**Table of Contents**

1. **Study-specific Methods Section for the Replication Section**
2. **Table S1.** Replication Results for C-Reactive Protein.
3. **Table S2.** Replication Results for lipids.
4. **Table S3.** Proxy variants for the Single Nucleotide Polymophisms not Available on the Metabochip Array.

**Study-Specific Methods Section for the Replication Section**

*LifeLines Cohort Study*

The LifeLines Cohort Study is a multi-disciplinary prospective population-based cohort study examining in a unique three-generation design the health and health-related behaviours of 165,000 persons living in the North East region of The Netherlands. It employs a broad range of investigative procedures in assessing the biomedical, socio-demographic, behavioural, physical and psychological factors which contribute to the health and disease of the general population, with a special focus on multimorbidity. In addition, the LifeLines project comprises a number of cross-sectional sub-studies, which investigate specific age-related conditions. These include investigations into metabolic and hormonal diseases, including obesity, cardiovascular and renal diseases, pulmonary diseases and allergy, cognitive function and depression, and musculoskeletal conditions. Written informed consent was obtained from every participant. All participants are between 18 and 90 years old at the time of enrolment. Recruitment has been going on since the end of 2006, and all subject have been included in the study. Blood was drawn in BD tubes anticoagulated with EDTA. C-reactive protein measurements were performed using an immune-nephelometric assay (Siemens BNII System) and an immunoturbidimetric assay (Roche Modular P, Mannheim, Germany). We included 12,838 subjects of which 7,470 were women.

The Illumina Cyto SNP12 v2 chip array was used to conduct genotyping. SNPs with a call rate <95%, MAF <0.01 and HWE p<10-4 were excluded. The final dataset comprised data on 257,581 SNPs in 13,395 of LifeLines participants. Imputation was conducted using the algorithm implemented in BEAGLE v3.1.0 with HapMap CEU release 24 (build 36) as a reference panel. Statistical analysis were done with PLINK.

*Rotterdam Study 2 and 3*

The Rotterdam study is a prospective population-based cohort study to investigate determinants of chronic diseases. In 2000, the second cohort of the Rotterdam Study (RS2) was started including 3,011 individuals aged 55 years and older who moved into the research area or had become 55. The cohort was further extended in 2006 with 3,932 individuals 45 years and older (RS3). The Rotterdam Study has been approved by the medical ethics committee according to the Population Screening Act: Rotterdam Study, executed by the Ministry of Health, Welfare and Sports of the Netherlands. All participants in the present analysis provided written informed consent to participate and to obtain information from their treating physicians.

The Illumina 550 duo was used to conduct genotyping in 2,612 individuals from RS2 and the Illumina 610 quad was used for genotyping in 3,540 individuals from RS3. Genotyping was successful in 2,272 and 3,361 individuals with a sample call rate >97.5% in RS2 and 3, respectively. SNPs with a call rate <95% and HWE p<10-6 were excluded in both cohorts. The final dataset comprised data on 537,405 SNPs in 2,157 RS2 participants and 543,360 in 3,054 RS3 participants. Imputation was conducted using the algorithm implemented in MACH. To obtain imputed data, more restrictive SNP filters including a minor allele frequency >0.01, SNP call rate >0.98, and HWE p-value >1×10-6 were applied and 466,389 passed the filters in RS2 and 514,073 in RS3. In total 2,543,887 SNPs were imputed using phased haplotypes of HapMap CEU trios in both RS2 and 3.

Fasting serum samples were collected at study baseline in both RS2 and 3. The samples were immediately put on ice and processed within 30 minutes, after which the samples were kept frozen at -20°C until the measurement of CRP in 2011-2012. High-sensitivity CRP was measured by use of Immunoturbidimetric assay (cobas^®^, Roche Diagnostics, Mannheim, Germany). In total, 1,943 paricipants from RS2 and 2,962 individuals from RS3 had both CRP measurements and genotyping available.

**Table S1**

| Single Nucleotide Polymorphism | Effect  Allele | Beta | Se | P-value | Heterogeneity I^2^ | Sample size |
| --- | --- | --- | --- | --- | --- | --- |
| *Significant replication* |  |  |  |  |  |  |
| rs1558902 | A | 0.050 | 0.012 | 2.7×10^-5^ | 64.2 | 17743 |
| rs10435719 | T | 0.059 | 0.014 | 2.6×10^-5^ | 0 | 15800 |
| rs7621025 | T | 0.043 | 0.014 | 1.4×10^-3^ | 12.5 | 17743 |
|  |  |  |  |  |  |  |
| *Non-significant replication* |  |  |  |  |  |  |
| rs12742376 | T | -0.007 | 0.033 | 0.83 | 0.0 | 17743 |
| rs17597773 | C | 0.006 | 0.013 | 0.66 | 60.8 | 17743 |
| rs8180991 | C | -0.018 | 0.015 | 0.21 | 0.0 | 17743 |
| rs1936797 | A | -0.003 | 0.013 | 0.81 | 42.0 | 17743 |
| rs2285810 | T | 0.007 | 0.012 | 0.58 | 9.0 | 12838 |
| rs6065906 | T | 0.012 | 0.015 | 0.39 | 44.3 | 17743 |
| rs661955 | C | -0.014 | 0.013 | 0.28 | 0.0 | 17743 |
| rs4703642 | A | 0.006 | 0.012 | 0.58 | 34.6 | 17743 |
| rs711752 | A | -0.013 | 0.022 | 0.56 | 57.3 | 4905 |
| rs17688076 | A | 0.016 | 0.022 | 0.45 | 0.0 | 17743 |
| rs2287921 | T | 0.000 | 0.012 | 0.98 | 0.0 | 17743 |
| rs11874381 | A | -0.003 | 0.023 | 0.89 | 0.0 | 4905 |
| rs1688043 | T | -0.064 | 0.041 | 0.12 | 0.0 | 4905 |
| rs2228603 | T | 0.013 | 0.023 | 0.58 | 40.7 | 17743 |
| rs2858310 | A | 0.033 | 0.014 | 0.02 | 0.0 | 17743 |
| rs469772 | T | -0.041 | 0.014 | 3.5×10^-3^ | 0.0 | 17743 |
| rs6951245 | A | -0.010 | 0.017 | 0.54 | 0.0 | 17743 |
| rs11220463 | A | -0.030 | 0.024 | 0.20 | 0.0 | 17743 |
| rs3822857 | C | -0.01 | 0.013 | 0.47 | 24.5 | 17743 |
| rs10761731 | A | -0.007 | 0.013 | 0.59 | 0.0 | 17743 |
| rs11887534 | C | -0.009 | 0.023 | 0.69 | 0.0 | 17743 |
| rs1529711 | T | 0.029 | 0.03 | 0.33 | 0.0 | 4905 |
| rs4871137 | T | 0.020 | 0.023 | 0.37 | 0.0 | 4905 |
| rs1077834 | T | -0.027 | 0.016 | 0.09 | 0.0 | 17743 |
| rs10744775 | T | 0.004 | 0.014 | 0.78 | 0.0 | 17743 |
| rs646776 | T | 0.031 | 0.026 | 0.23 | 0.0 | 4905 |
| rs1127311 | A | -0.028 | 0.011 | 0.01 | 68.7 | 17743 |
| rs10832027 | A | 0.011 | 0.012 | 0.36 | 60.5 | 17743 |
| rs9378212 | T | 0.020 | 0.015 | 0.19 | 0.0 | 12838 |
| rs1441759 | C | -0.119 | 0.106 | 0.26 | 45.9 | 4905 |
| rs2277844 | A | -0.015 | 0.012 | 0.20 | 0.0 | 17743 |
| rs571312 | A | 0.032 | 0.013 | 0.01 | 0.0 | 17743 |
| rs174546 | T | -0.007 | 0.016 | 0.68 | 0.0 | 17743 |

P-value for significant replication was 1.4×10^-3^ for CRP.

Se denotes standard error.

**Table S2**

| Single Nucleotide Polymorphism | Effect  Allele | Beta | Se | P-value |
| --- | --- | --- | --- | --- |
| *LDL-cholesterol* |  |  |  |  |
| rs10186133* | G | 0.003 | 0.005 | 0.58 |
| rs1800961 | C | 0.066 | 0.013 | 6.1×10^-6^ |
| rs340025* | T | 0.006 | 0.005 | 0.25 |
| rs676388* | C | 0.025 | 0.005 | 7.2×10^-6^ |
|  |  |  |  |  |
| *HDL-cholesterol* |  |  |  |  |
| rs10761741* | T | 0.015 | 0.005 | 5.3×10^-3^ |
| rs12194148* | T | 0.017 | 0.006 | 0.01 |
| rs12742376 | C | 0.058 | 0.008 | 2.1×10^-10^ |
| rs1558902 | T | 0.015 | 0.005 | 5.3×10^-3^ |
| rs1936797 | A | 0.022 | 0.005 | 7.1×10^-5^ |
| rs4871137 | G | 0.016 | 0.005 | 2.4×10^-3^ |
| rs7621025 | T | 0.034 | 0.006 | 2.2×10^-8^ |
|  |  |  |  |  |
| *Triglycerides* |  |  |  |  |
| rs10832027 | A | 0.014 | 0.005 | 7.2×10^-3^ |
| rs11998678* | C | 0.020 | 0.005 | 8.5×10^-5^ |
| rs16842484* | C | 0.006 | 0.005 | 0.50 |
| rs2686555 | G | 0.004 | 0.005 | 0.42 |
| rs4660293* | G | 0.017 | 0.006 | 4.2×10^-3^ |
| rs523288* | T | 0.001 | 0.006 | 0.60 |
| rs6731551* | T | 0.002 | 0.005 | 0.80 |
| rs7539471* | G | 0.000 | 0.005 | 0.74 |
| rs9987289 | A | 0.030 | 0.009 | 2.9×10^-3^ |
|  |  |  |  |  |
| *Total cholesterol* |  |  |  |  |
| rs1997243* | G | 0.030 | 0.007 | 1.9×10^-5^ |
| rs340025 | C | 0.003 | 0.005 | 0.60 |
| rs577272 | A | 0.014 | 0.005 | 6.1×10^-3^ |

*Proxy SNPs for the pleiotropic SNPs that were not available on the Metabochip, as described in Supplementary Table III.

P-value for significant replication was 2.2×10^-3^ for lipids.

Se denotes standard error.

**Table S3**

| SNP | Proxy | LD (R^2^) |
| --- | --- | --- |
| *LDL-cholesterol* |  |  |
| rs12711751 | rs10186133 | 0.65 |
| rs340005 | rs340025 | 0.80 |
| rs2287921 | rs676388 | 0.68 |
|  |  |  |
| *HDL-cholesterol* |  |  |
| rs10761731 | rs10761741 | 0.97 |
| rs9378212 | rs12194148 | 1.00 |
|  |  |  |
| *Triglycerides* |  |  |
| rs10435719 | rs11998678* | 0.51 |
| rs12755606 | rs16842484* | 0.57 |
| rs4660808 | rs4660293* | 1.00 |
| rs571312 | rs523288* | 1.00 |
| rs13409360 | rs6731551* | 0.70 |
| rs11208722 | rs7539471* | 0.90 |
|  |  |  |
| *Total cholesterol* |  |  |
| rs6951245 | rs1997243* | 1.00 |
